# Identification, Cloning and Expression of Ferritin M-like Subunit from the Indian Oyster, *Magallana bilineata* (Röding, 1798)

**DOI:** 10.3390/genes17030330

**Published:** 2026-03-18

**Authors:** Esha Arshad, Mangottil Ayyappan Pradeep, Gokhlesh Kumar, Nikathil Raveendranathan Dhanutha, Eranezhath Ashok Nisha, Thevanattil Sairanksha Azhar Shahansha, Koyadan Kizhakkedath Vijayan

**Affiliations:** 1 Marine Biotechnology, Fish Nutrition and Health Division, Indian Council of Agricultural Research—Central Marine Fisheries Research Institute, Kochi 682018, India; 2 Faculty of Marine Sciences, Lakeside Campus, Cochin University of Science and Technology, Kochi 682016, India

**Keywords:** Indian backwater oyster, ferritin, M type subunit, iron-responsive element, Mbi-Fer

## Abstract

**Background/Objectives**: Ferritins are key iron-sequestering proteins that maintain cellular homeostasis by storing iron in a bioavailable and nontoxic form. They also contribute to innate immunity, cellular proliferation and differentiation, shell formation, and protection against oxidative stress. In this study, we identified and characterized the M-type subunit of ferritin (Mbi-Fer) from the Indian backwater oyster, *Magallana bilineata* (Röding, 1798). **Methods**: A full-length cDNA of *Mbi-Fer* was sequenced and analyzed, and its gene expression was quantified in oysters collected from their natural habitat. Additionally, the coding region of *Mbi-Fer* was transformed and expressed in *Escherichia coli*, and the recombinant protein was purified and analyzed. **Results**: Mbi-Fer exhibited all the typical features of M-type ferritins, including the ferroxidation site of the H subunit and the nucleation core of the L subunit. The amino acid sequence alignment and phylogenetic analysis showed high similarity to the M-type ferritin subunits of *Magallana gigas* (Thunberg, 1793). A putative iron-responsive element was identified in the 5′ UTR, indicating potential post-transcriptional regulation. *Mbi-Fer* expression in wild oysters was increased by more than fourfold, relative to laboratory-maintained control oysters. The recombinant expression result revealed a unique protein band that was specific to a ferritin M-like subunit, with an approximately molecular weight of 20 kDa. **Conclusions**: Our findings suggest that Mbi-Fer may play a role in both the iron storage and shell formation of backwater oysters and may serve as a valuable molecular marker of oxidative and environmental stress responses in estuarine bivalves.

## 1. Introduction

Ferritins are members of the di-iron carboxylate protein superfamily and primarily function in iron storage and detoxification [1,2]. They are ubiquitous and highly conserved proteins that play a crucial part in cellular iron metabolism by storing iron in bio-available and non-toxic forms [3,4]. Ferritin contributes to cellular homeostasis and protects cells from oxidative damage by sequestering excess iron, which would otherwise be oxidized to the ferric state, generating harmful reactive oxygen species [5]. Ferritin originated early in evolution and is found across all kingdoms of life, including bacteria, plants, and animals [2,6]. It was first discovered in the horse spleen [7], and its smooth isolation is facilitated by several distinct biochemical characteristics, such as stability at high temperatures (80 °C), relative insolubility in ammonium sulfate, and its ability to crystallize with cadmium salts [8,9]. Currently, more than 7000 ferritin sequences and fragments from over 900 species are available in gene databases [10].

In vertebrates, functional ferritin self-assembles into a hollow, spherical, cage-like structure composed of 24 polypeptide subunits with octahedral (432) symmetry, providing storage capacity for ferric iron. Each polypeptide subunit folds into a four-helical bundle. The assembled protein has a molecular mass of approximately 450 kDa and can store up to 4500 iron atoms in its mineral core [11,12]. The three-dimensional structure of ferritin is highly conserved among prokaryotes and eukaryotes, which is essential for their iron storage and transport functions. In higher eukaryotes, two major ferritins, namely, heavy (H) and light (L) chains, are encoded by distinct genes and co-assemble to form functional heteropolymers [2,11]. The H-chain contains a ferroxidase center that oxidizes ferrous (Fe^2+^) to ferric (Fe^3+^) iron, while the L-chain lacks this site but contributes a nucleation center that is crucial for mineralization. Some lower vertebrates, including fish and amphibians, also express a third type the M-chain, which combines the ferroxidase activity of the H-chain with the mineralization features of the L-chain [13,14].

Although ferritins possess conserved sequence and structural characteristics, they vary among organisms in size, cellular and subcellular distribution, and regulatory patterns of synthesis [1,3]. Ferritin molecules are classified into three subgroups: cytosolic, secretory, and mitochondrial [11]. Cytosolic ferritins are mainly involved in intracellular iron storage and homeostasis. Secretory ferritins function as iron transport or donor molecules and may play an important role in exporting iron from the cell. Mitochondrial ferritin serves an antioxidant role by protecting mitochondria against iron-induced oxidative damage [3,15,16,17].

Ferritins and coding genes have been extensively studied in mammals and insects. Molluscan ferritins have been reported since the 1970s [18,19,20], but the available data remain fragmented. Around 60 ferritin subunit genes have been reported across molluscan species, with most showing sequence similarity to the H-type subunit [21]. The expression of both secretory and non-secretory ferritin subunits is unique to mollusks: a feature rarely observed in other animal groups [13]. Among bivalve molluscs, ferritin subunits have been identified and characterized in several species such as the Pacific oyster, *Crassostrea gigas* (Thunberg, 1793) [21], pearl oyster, *Pinctada fucata* (Gould 1850) [9], bay scallop, *Argopecten irradians* (Lamarck, 1819) [22], Asiatic hard clam, *Meretrix meretrix* (Linnaeus, 1758), Chinese razor clam, *Sinonovacula constricta* (Lamarck, 1818), Manila clam, *Ruditapes philippinarum* (Adams & Reeve, 1850) [23,24,25], Biwa pearl mussel, *Hyriopsis schlegelii* (Martens, 1861) [26], the Asian green mussel (*Perna viridis*) [27], and red abalone *Haliotis rufescens* (Swainson, 1822) [28].

In mollusks, ferritins are involved in diverse biological processes beyond iron metabolism. These include contributions to innate immunity [21,29,30]; cellular proliferation and differentiation [31,32]; shell formation [9]; iron supply to iron-dependent molecules, such as nitrogenase and ribonucleoreductase [2]; and protection against oxidative stress [33]. Their expression is upregulated by a range of stimuli, including pathogens [23,26,34,35,36], pH [37], iron [38,39], poly (I:C) [39], and oxidative stress [9].

*Magallana bilineata* (Röding, 1798), commonly known as the Indian backwater oyster, is an ecologically and commercially important aquaculture species in India and the Philippines. It is an edible oyster that is widely distributed along the Indian coastline, particularly in estuarine and backwater regions [40]. Recently, the first reference genome for *M. bilineata* was sequenced and assembled to support oyster conservation and aquaculture [41]. Ferritin is the primary protein responsible for iron storage and metabolism in nearly all organisms; however, the identification and characterization of M-type ferritin have not yet been explored in the Indian backwater oyster.

The digestive tissue of the oyster is known to be rich in ferritin and is involved in key physiological processes, making it a particularly relevant organ for studying iron metabolism and stress-related responses [28]. Therefore, the objective of this study was to identify and functionally characterize the M-type ferritin subunit (Mbi-Fer) from the digestive tissue of *M. bilineata*. We sequenced and analyzed the full-length cDNA of *Mbi-Fer* and quantified its gene expression in wild-habitat oysters and performed recombinant expression and purification to further characterize the protein.

## 2. Materials and Methods

### 2.1. Oyster Collection and Maintenance

**Figure 1:**
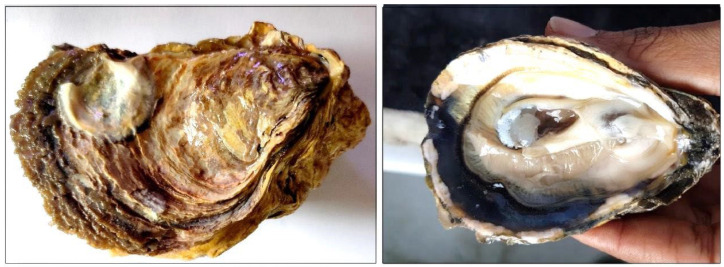
*M. bilineata*, Indian backwater oyster. The image on the left shows the outer shell view of an adult *M. bilineata*. The image on the right shows the oyster with its shells opened.

Adult *M. bilineata* oysters (Figure 1) were randomly collected from wild populations in the Sathar Island region of the Kodungallur-Azhikode estuary (10°11′26.34″ N and 76°11′28.88″ E), Ernakulam, Kerala, India (Supplementary Figure S1) during the pre-monsoon period month of May. All procedures were performed in compliance with the institute guidelines on marine animal research. A total of 24 oysters were divided into two groups: wild habitat oysters and laboratory-maintained oysters. Twelve wild oysters were immediately anesthetized with benzocaine (1.2 gm per liter). Individuals were then sampled and their digestive tissues were preserved in RNAlater (Thermo Fisher Scientific, Waltham, MA, USA) and stored at −80 °C for molecular analysis. The remaining 12 oysters were transported to the wet laboratory at the institute hatchery, thoroughly cleaned to remove grit and algae, and maintained under controlled conditions.

During acclimatization, the oysters were held in aerated aquaria containing filtered seawater (salinity 22 ppt and temperature 25–28 °C). Oysters were fed daily with a mixture of live cultures of *Isochrysis galbana* (50%) and *Nannochloropsis salina* (50%), except on the day prior to sampling. After the 7 day acclimatization period, the laboratory-maintained control oysters were anesthetized using the same protocol as above and their digestive tissues were sampled and preserved in RNAlater. The 7 day acclimatization period was selected based on evidence that oysters rapidly adjust to laboratory conditions and that major stress-related and metabolic processes stabilize within this time frame. Additionally, oysters were examined for the presence of *Perkinsus* spp., using *Perkinsus* genus-specific PCR primers [42], where they were found to be free of Perkinsus disease. Environmental parameters were recorded for both wild-collection sites and laboratory control conditions, using a salinometer and a precision thermometer, respectively. pH and turbidity were determined in the laboratory, using a pH meter (Cyberscan PC5500, Eutech Instruments, Singapore) and a digital turbidity meter (Micro1000 IR, HF Scientific, Fort Myers, FL, USA). Dissolved oxygen (DO) concentrations were measured using the modified Winkler titration method. Nutrient parameters, including chlorophyll-a, ammonia–nitrogen (NH_4_^+^–N), nitrate–nitrogen (NO_3_^−^–N), nitrite–nitrogen (NO_2_^−^–N), and phosphate–phosphorus (PO_4_^3−^–P), were analyzed following the standard spectrophotometric protocols.

### 2.2. Total RNA Extraction

The total RNA was isolated from the digestive tissues of both wild habitat and laboratory-maintained oysters using TRIzol reagent (Thermo Fisher Scientific, USA), followed by DNase treatment according to the manufacturer’s protocol. The integrity and purity of total RNA was assessed using a spectrophotometer, BioPhotometer plus (Eppendorf, Hamburg, Germany), and further confirmed by electrophoresis on a 1.5% agarose gel. Poly(A)+ mRNA was subsequently purified using the Oligotex mRNA Mini Kit (Qiagen, Hilden, Germany).

### 2.3. Cloning of Mbi-Fer

The sequence of *Mbi-Fer* (MH887445.1) was obtained from our earlier study [43], in which a suppression subtractive hybridization cDNA library was constructed from the digestive tissues of *M. bilineata*. To generate the full-length sequence of *Mbi-Fer*, RACE-PCR was performed in both the 5′ and 3′ directions to obtain a complete reading frame using the SMARTer RACE cDNA amplification kit (Takara Bio Inc, Kusatsu, Japan). Both gene-specific RACE primers (ferritin 3′ RACE primer: 5′GCGGACGGTCACAAGGATGCACAGATGT3′ and ferritin 5′ RACE primer: 5′GAACTTGCTGAATCCAGGGAGGGCGACA3′) were designed using primer3 plus software version: 3.3.0 (https://www.primer3plus.com/) and synthesized by Sigma-Aldrich, India.

A total of 1 µg RNA was used for RACE-PCR, employing the protocol recommended by the manufacturer (Takara Bio Inc, Kusatsu Japan). Both 3′ and 5′ RACE ready cDNA were synthesized, and the RACE reactions were performed using the gene-specific primer paired with a RACE universal primer in a 20 µL reaction mixture, in the following step-down protocol: 5 cycles of 94 °C for 30 s and 72 °C for 3 min; 5 cycles of 94 °C for 30 s, 70 °C for 30 s, and 72 °C for 3 min; and 27 cycles at 94 °C for 30 s, 68 °C for 30 s, and 72 °C for 3 min. The amplified products were ligated and cloned into the pJET1.2 cloning vector (Thermo Fisher Scientific, Waltham, MA, USA) and transformed into top 10 competent cells. The transformed cells were plated on Luria broth (LB) agar containing ampicillin (100 µg/mL) for selection. Colonies harboring inserts of the expected size were screened by colony PCR, using both vector- and gene-specific primers. Positive clones were cultured overnight in LB supplemented with ampicillin (100 μg/mL). Plasmids were extracted from these overnight cultures and sequenced using pJET vector-specific primers to confirm the identity of the insert.

### 2.4. Sequence Analysis

The 3′ and 5′ sequences obtained were aligned, after removing the vector sequences, using the Lasergene assembly program (Seqman, Madison, WI, USA). The trimmed 3′ and 5′ sequences were aligned with the previously obtained gene segments to generate a single contig with overlapping segments. The contig was then analyzed using NCBI BLAST software version 2.15.0 for comparison with known cDNAs, as well as for the identification of orthologs. The open reading frame of Mbi-Fer was determined and the amino acid sequence was predicted using EditSeq in Lasergene software version 17.1. The software was also used to predict the molecular weight and pI value of the ferritin protein. The motif sequences were predicted using InterProScan software version 5.77-108 (https://www.ebi.ac.uk/interpro/search/sequence/) and PROSITE version 20.124 (https://prosite.expasy.org/). 

Signal peptide sequences were predicted using SignalP version 4.1 (https://services.healthtech.dtu.dk/services/SignalP-4.1/) and potential glycosylation sites were determined using the NetNGlyc software version 1.0 (https://services.healthtech.dtu.dk/services/NetNGlyc-1.0/). The tertiary structure of Mbi-Fer was predicted using the SWISS-MODEL algorithm version 2020 (http://swissmodel.expasy.org/).

### 2.5. Phylogenetic Analysis

Twenty-seven different vertebrate and invertebrate ferritin genes and subunits were obtained from the NCBI protein database, along with the *Mbi-Fer* identified in this study. Among the sequences used in the analysis, twenty belonged to representative mollusk species. The GenBank accession numbers for the ferritin sequence designations were as follows: *M. gigas* (ferritin GF1, AAP83793.1; ferritin GF2, AAP83794.1; ferritin, CAD91440.1; soma ferritin, CAD92096.1; soma ferritin, EKC30759.1; yolk ferritin, EKC42967.1; ferritin, lower subunit, EKC42968.1), *M. ariakensis* (ferritin, ACU25551.1), *P. fucata* (ferritin-like protein, AAQ12076.1), *M. meretrix* (ferritin subunit, AAZ20754.1), *S. constricta* (ferritin, ACZ65230.1), *H. diversicolor* (ferritin, ABY87353.1), *H. discus discus* (ferritin subunit 1, ABG88845.1; ferritin subunit 2, ABG88846.1), *R. philippinarum* (ferritin subunit, ADX31290.1), *Ostrea edulis* (Linnaeus, 1758) (ferritin, AFK73708.1), *Aplysia californica* (Cooper, 1863) (soma ferritin, NP_001191661.1), *Reishia clavigera* (Küster, 1860) (ferritin, AET43963.1), *Concholepas concholepas* (Bruguière, 1789) (ferritin, AGC81883.1), *Conus ermineus* (Born, 1778) (ferritin, AXL95451.1), *Danio rerio* (Hamilton, 1822) (ferritin heavy chain, NP_571660.1), *Ictalurus punctatus* (Rafinesque, 1818) (ferritin heavy subunit, AAY86949.1), *Salmo salar* (Linnaeus, 1758) (ferritin heavy subunit, NP_001117129.1), *Equus caballus* (Linnaeus, 1758) (ferritin heavy chain, NP_001093883.1), *Mus musculus* (Linnaeus, 1758) (ferritin heavy chain, NP_034369.1; ferritin light chain 1, NP_034370.2), and *Homo sapiens* (Linnaeus, 1758) (FTH1 partial protein, AAH70494.1). A multiple sequence alignment was generated using homologous ferritin gene sequences from other species, obtained from the NCBI BLAST query using the Bio-Edit multiple alignment tool and ClustalW [44]. Subsequently, a phylogenetic tree was generated using MEGA version 11 software with the neighbor-joining method, using a bootstrap algorithm with 500 replicates [45].

### 2.6. Quantitative Real Time-PCR

To compare ferritin expression between wild and laboratory-maintained control oysters, quantitative RT-PCR was performed. The RNA samples were reverse-transcribed into cDNA using the iScript™ cDNA Synthesis Kit (Bio-Rad, Hercules, CA, USA). *Mbi-Fer*-specific real time primers 5′ ACGCCTTGACTCCTAAAGCC 3′ (Ferritin-F) and 5′ CCTCCGTTACCGATTCCCAA 3′ (Ferritin-R) were designed using the Primer3Plus platform. Each 20 µL qPCR reaction contained 10 µL of iQ™ SYBR^®^ Green Supermix 2X (Bio-Rad), 1 µL of diluted cDNA (1:20), 0.5 µM of each primer, and nuclease-free water. Reactions were performed on a LightCycler 96 (Roche, Basel, Switzerland)), using the following thermal profile: initial denaturation at 94 °C for 10 min; 40 cycles of 94 °C for 10 s and 60 °C for 30 s; followed by a melting curve analysis from 99 °C to 60 °C with a 0.5 °C decrement every 10 s to verify amplicon specificity. All reactions were run in triplicate for each biological replicate. Data were normalized to the reference 18S rRNA gene [46]. The relative ferritin gene expression was quantified using the Pfaffl method [47]. The statistical significance was assessed using a two-tailed Student’s *t*-test, with *p* < 0.05 considered significant.

### 2.7. Recombinant Cloning, Expression, and Purification of Mbi-Fer

The coding region of the *Mbi-Fer* was amplified using the oligonucleotide forward and reverse primers containing BspH1 and SalI restriction sites: 5′ TCTTATTTCATGAGCGCTGAATCCCAATGTCGCCAGA3′ (Ferritin_BspH1_F) and reverse primer 5′ ATTTAATAGTCGACGGAGTCGAGGC GTCGGTCGTAC 3′ (Ferritin_SalI_R). The obtained PCR product was double digested with BspH1 and SalI restriction enzymes (New England Biolabs, Ipswich, MA, USA). The digested PCR product was purified using a MinElute PCR purification kit (Qiagen, Hilden, Germany) and cloned into the NcoI and XhoI restriction sites of the pET28b expression vector (Novagen, Darmstadt, Germany). The resulting recombinant plasmid (pET28b-*Mbi-Fer*) was transformed into *Escherichia coli* BL21 cells. The transformed cells were plated on LB agar containing kanamycin (50 µg/mL) for selection. Positive clones were cultured in LB supplemented with kanamycin (50 µg/mL) at 37 °C, with continuous shaking at 225 rpm. At O.D._600_, approximately equal to 0.6, the expression of *Mbi-Fer* was induced, using 0.6 mM isopropyl-β-D-thiogalactopyranoside (IPTG). Following induction, the cultures were incubated for 4 h at 37 °C with shaking. The induced and uninduced cultures were harvested by centrifugation at 8000 rpm for 5 min. The expressed Mbi-Fer was purified by affinity chromatography, using a Ni-NTA affinity column (Cytiva, Uppsala, Sweden) under denaturing conditions, according to the manufacturer’s instructions. The purified proteins were separated and analyzed by 12% sodium dodecyl sulfate polyacrylamide gel electrophoresis (SDS-PAGE).

## 3. Results

### 3.1. Environmental Parameters

The environmental parameters of the wild habitat site and the laboratory-maintained control oysters are given in Table 1. The water temperature and salinity at the wild collection site were 31 °C and 15 ppt, respectively, whereas laboratory-maintained aquaria conditions were maintained at 28 °C and 22 ppt to ensure optimal oyster growth. Additionally, dissolved oxygen levels were higher in the laboratory-maintained aquaria compared to the wild habitat, whereas turbidity and nutrient concentrations were elevated in the natural environment, which was characteristic of tropical estuarine systems. Nutrient levels in the laboratory aquaria were below detectable limits.

### 3.2. Sequence Analysis of Mbi-Fer

**Figure 2:**
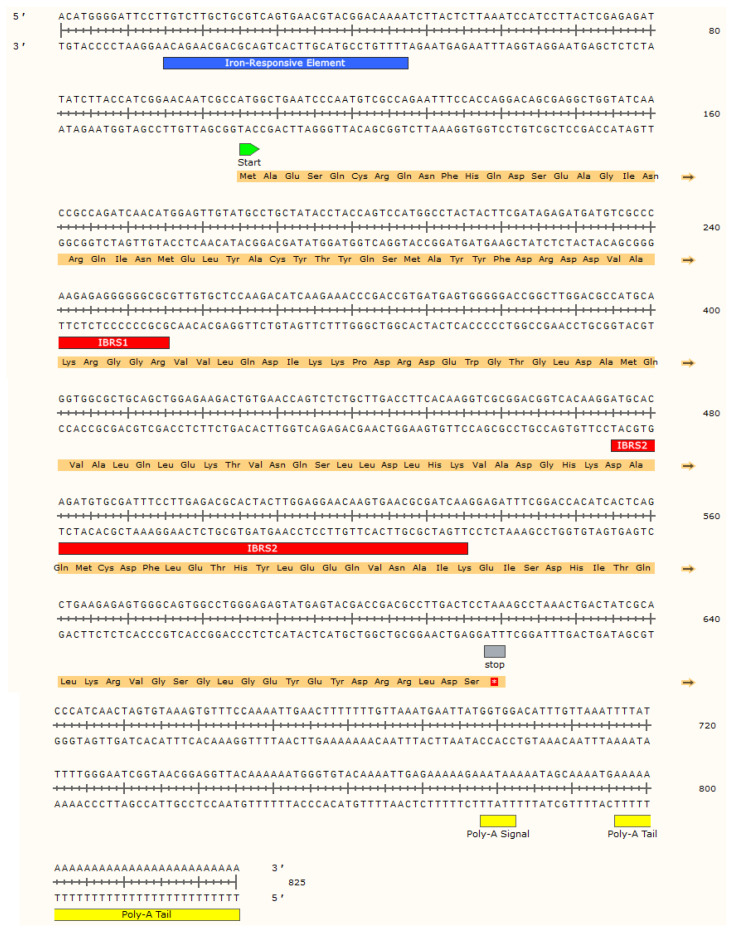
Full length nucleotide and deduced amino acid sequence of *M. bilineata* ferritin (Mbi-Fer). Iron-responsive element (IRE) highlighted in blue and putative ferritin iron-binding region signature 2 (IBRS2) in red. The poly-A signal and tail are at the end, in yellow.

The full-length sequence of *Mbi-Fer* was 825 bp, comprising a 104 bp in the 5′ untranslated region and a 205 bp in the 3′ untranslated region, including a poly(A) tail. The open reading frame (ORF) was 516 bp in length. The deduced amino acid sequence of the coding *Mbi-Fer* consisted of 171 residues, with a predicted molecular weight of 20 kDa. The full-length cDNA sequence and the deduced amino acids of *Mbi-Fer* are shown in Figure 2. The predicted pI value of the putative protein was 5.224, consisting of 22 strongly basic (+) amino acids (K and R), 31 strongly acidic (−) amino acids (D and E), 47 hydrophobic amino acids (A, I, L, F, W, and V), and 46 polar amino acids (N, C, Q, S, T, and Y). BLASTp analysis of the deduced amino acid sequence showed 97.66% similarity to the ferritin of *M. gigas* (AAP83793.1), 85.38% similarity to *P. fucata* (AAQ12076.1), and 80.84% similarity to *H. diversicolor* (ABY87353.1).

### 3.3. Iron-Responsive Element

**Figure 3:**
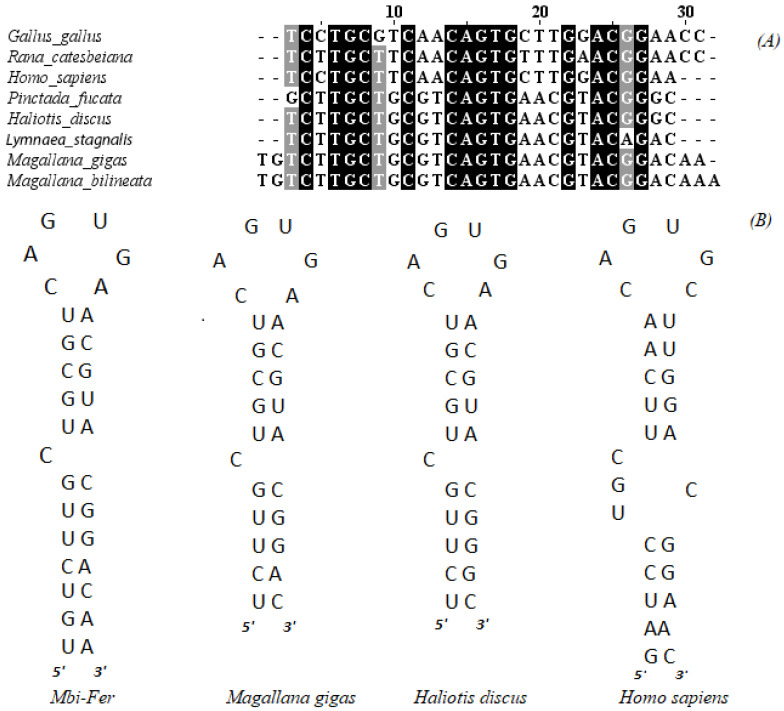
Alignment of IRE and stem-loop structure of *Mbi-Fer* with that of different species. (**A**) Alignment of *Mbi-Fer* IRE sequence with that of other selected ferritins. Identical residues are shaded in black and consensus residues are shaded in gray. (**B**) A comparison of the IRE stem loop structure of *Mbi-Fer* and known ferritins from *M. gigas* (AY321299.1), *H. discus discus* (DQ821494.1) and *H. sapiens* (BC070494.1).

A key characteristic feature of all ferritins is the presence of an iron-responsive element (IRE) in the 5′ untranslated region (UTR) of the gene [48]. A putative IRE was also detected in *Mbi-Fer* at position 15 bp, and 57 bp upstream of the start codon. The IRE contains a conserved loop sequence 5′-CAGUGN-3′ and a bulged “C”, located six nucleotides upstream of this loop. A comparison of the IRE stem-loop structure and sequence of *Mbi-Fer* with those of other known ferritins is shown in Figure 3.

Analysis of the *Mbi-Fer* IRE sequence showed the highest identity (100%) with the IRE of ferritin GF1 from *M. gigas* (AY321299.1). Additionally, it shared 96.3% similarity with the respective IRE sequences of *Lymnaea stagnalis* (Linnaeus, 1758) (X56778.1) and *H. discus discus* (DQ821494.1), 93% with *P. fucata* (AF547223), 67% with *H. sapiens* heavy chain subunit (BC070494.1), 65.52% with *Rana catesbeiana* (Shaw, 1802) (M12120.1), and 58.62% with the IRE of *Gallus gallus* (Linnaeus, 1758) heavy chain (NM_205086).

### 3.4. Protein Structure of Mbi-Fer

Mbi-Fer contains several conserved amino acid residues and signature motifs that are characteristic of all ferritins. These included two putative iron-binding region signatures, IBRS1 (59EEREHAEKLMKYQNKRGGR77) and IBRS2 (124DAQMCDFLETHY LEEQVNAIK144), as predicted by the PROSITE program. Seven amino acid residues (i.e., 25E, 32Y, 59E, 60E, 63H, 105E, and 139Q), known as metal ligands that are part of the ferroxidase site involved in the catalysis of iron (II) oxidation, were found to be conserved in Mbi-Fer. In addition, three amino acid residues (58D, 59E, and 62E) that promote ferrihydrite nucleation and another three amino acid residues (116H, 129D, and 132E) of an ion channel that allows the ferritin gene to access iron ions were also conserved in Mbi-Fer. This also has a potential N-glycosylation site (N, Q, and S) at a location from amino acids 109 to 111. No signal peptide was detected at the N-terminus of the protein.

**Figure 4:**
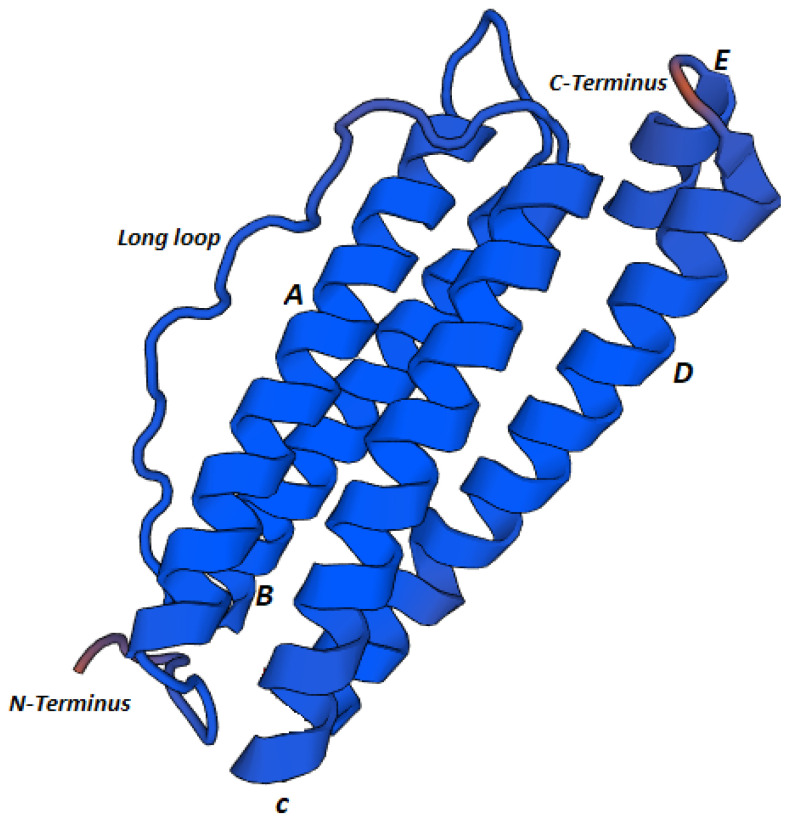
Predicted three-dimensional structure of Mbi-Fer using SWISS-MODEL. The model revealed four long α-helices (A–D) originating from the N-terminus, arranged in parallel and connected by random coils and turns. A fifth short α-helix (E) was observed at the C-terminus.

Tertiary structure predictions of Mbi-Fer revealed the typical architecture of ferritin proteins. Mbi-Fer displayed a simple structure consisting of five α-helices: four long helices and one short helix at the C-terminal end. The helices are designated A to E, beginning at the N-terminus, with helix E being the shortest. Helices B and C are connected by a long loop. The three-dimensional structure of Mbi-Fer (Figure 4) was modeled using the ferritin structure of *Chaetopterus variopedatus* (Renier, 1804) (PDB ID: 5wpn.1.A) as a template.

### 3.5. Phylogenetic Analysis of Ferritin

**Figure 5:**
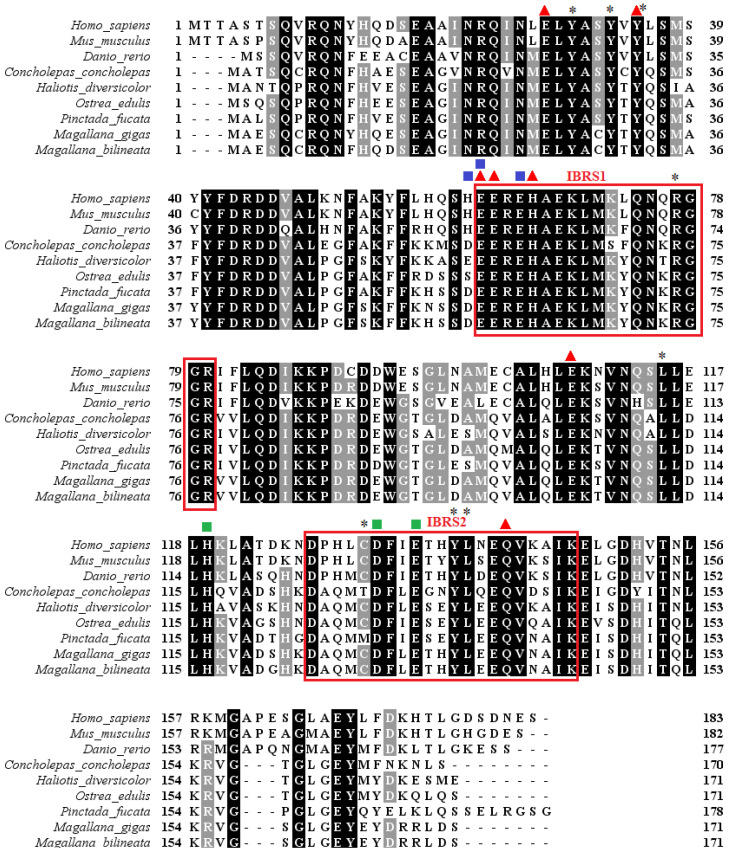
Multiple amino acid sequence alignment of Mbi-Fer with other species of ferritin. The two-signature iron-binding regions are boxed. The seven metal binding ligands are indicated in ▲, the three residues of ferrihydrite nucleation in ∎ and the three residues of the iron ion channel are indicated in ∎. All other conserved residues are shown by an “*”. The amino acid sequence data were acquired from the GenBank database and the accession numbers are as follows: *H. sapiens* (AAH70494.1), *M. musculus* (NP_034369.1), *D. rerio* (NP_571660.1), *C. conchlepas* (AGC81883.1), *H. diversicolor* (ABY87353.1), *O. edulis* (AFK73708.1), *P. fucata* (AAQ12076.1), and *M. gigas* (AAP83793.1).

**Figure 6:**
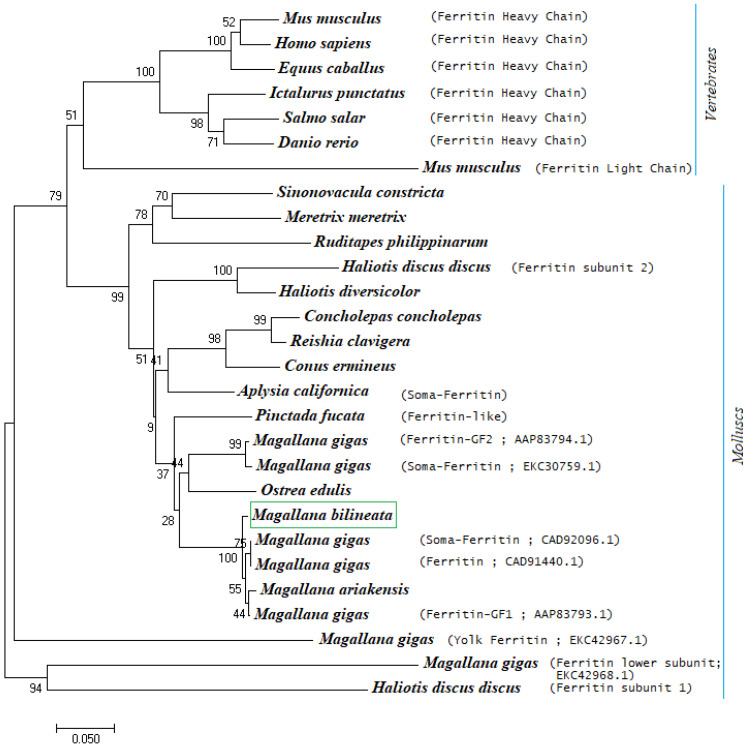
Phylogenetic analysis of *Mbi*-Fer. The tree is constructed by the neighbor-joining method, using the MEGA program, based on the clustalW algorithm.

A multiple sequence alignment of *Mbi-Fer* with ferritin sequences retrieved from the NCBI database showed that *Mbi-Fer* shares a high identity with its invertebrate orthologs. The iron-binding signature motif-1 (IBRS1) was found to be highly conserved across both vertebrates and invertebrate species. The deduced amino acid sequence of *Mbi-Fer* showed the highest similarity (97.66%) to ferritin GF1 of *M. gigas* (AAP83793.1), followed by 85.38% similarity to a ferritin-like protein of *P. fucata* (AAQ12076.1). In comparison with vertebrate ferritins, *Mbi-Fer* shared 66.08%, 63.53%, and 63.16% similarity with ferritin heavy chain of *H. sapiens* (AAH70494.1), *D. rerio* (NP_571660.1), and *M. musculus* (NP_034369.1), respectively. Table 2 summarizes the sequence similarity of *Mbi-Fer* with other ferritin sequences. The multiple sequence alignment of *Mbi-Fer* with ferritins from other species is demonstrated in Figure 5. The phylogenetic tree was resolved into two main clusters: the vertebrate clade and invertebrate molluscan clade. *Mbi-Fer* forms part of the subclade consisting of oyster ferritins and showed significant similarity with ferritin sequences from *M. gigas* and *M. ariakensis* (Figure 6).

### 3.6. Ferritin Expression

**Figure 7:**
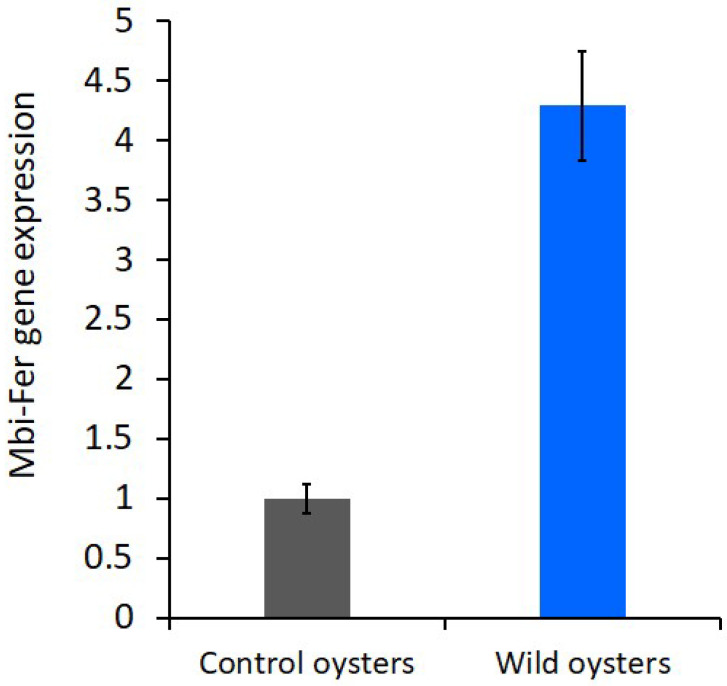
Relative ferritin expression in laboratory-maintained control oysters and wild habitat oysters. Expression levels were normalized to 18S rRNA. Bars represent mean fold change ± SD from twelve biological samples (*p* < 0.001).

The laboratory-maintained control oysters group showed baseline *ferritin* expression (1.00 ± 0.04), whereas the wild oysters exhibited a 4.34 ± 0.35-fold upregulation (Figure 7). Statistical analysis of the gene expression data confirmed that this difference was highly significant (*p* < 0.001). The marked upregulation of ferritin in wild oysters indicates a strong stress-responsive induction, likely triggered by environmental conditions such as higher temperature, reduced salinity, and increased turbidity compared to laboratory-maintained control oysters.

### 3.7. Recombinant Protein

As expected, Mbi-Fer bands were observed at approximately 20 kDa. When analyzed by SDS-PAGE under reducing conditions, the purified Mbi-Fer migrated as a single band of approximately 20 kDa (Supplementary Figure S2), consistent with the predicted molecular size of the ferritin protein.

## 4. Discussion

Genetic diversity in oysters has been extensively studied because of its basic role in adaptation to environmental challenges. Research on *Crassostrea angulata* (Lamarck, 1819) and *C. gigas* (Thunberg, 1793) has shown significant genetic diversity, both between populations and within populations, using microsatellite markers, *Cox1 Gene* sequencing and phylogenetic analysis [49,50]. Recent genome-wide SNP data from *M. bilineata* confirmed genetic diversity between Sri Lankan and Fijian sampling locations [41]. Such genetic variability may underlie differences in environmental conditions and metal homeostasis, which underscores the need to investigate key stress-related genes, particularly ferritins, in oysters.

Ferritins have been reported from many different organisms, and studied in detail regarding their structure, function, gene regulation, and evolution significance. These active molecules play essential roles in regulating iron availability within cells and preventing cellular oxidative damage. Consequently, ferritin is found in nearly all cell types and compartments within an organism. Despite sharing common structural and sequence features, ferritins vary across species in terms of molecular size, cellular or subcellular localization, and regulatory mechanisms [27]. Animals such as mollusks are known to encode for more than a single type of ferritin [13], and various functions have been suggested for molluscan ferritins, such as their role in defense, shell formation, larval development, and protecting cells from their environmental stressors. Although some ferritin genes from mollusks have been described, detailed knowledge on their properties and roles is limited, particularly for the Indian backwater oyster, *M. bilineata*, for which no information on ferritin isoforms is currently available. In this study, we focused on the digestive tissues of *M. bilineata* because this organ serves as a major site for iron storage and plays key roles in various metabolic processes, including digestion, macromolecule degradation, gas exchange, and the accumulation and detoxification of xenobiotics [28,51,52,53]. These physiological features make the digestive tissue an appropriate and biologically relevant target for investigating the molecular characteristics and functional roles of the M-type ferritin in this species.

We successfully identified and characterized the full-length ferritin cDNA of *Mbi-Fer* from the digestive tissues of *M. bilineata*. The amplified *Mbi-Fer* contains an ORF of 516 bp, encoding a polypeptide of 171 amino acid residues, which is consistent with the typical size range observed in most molluscan ferritins [13,21,24]. QRT-PCR analysis revealed a significant upregulation (~4.3-fold) of *Mbi*-Fer expression in wild-collected oysters, relative to laboratory-maintained control oysters. Studies on oysters and other bivalves have shown that under various environmental conditions, genes linked to ferritin and antioxidants are upregulated. For instance, in the thick-shell mussel, *Mytilus coruscus* (Linnaeus, 1758) exposed to environmental marine pollution benzo[a]pyrene, qRT-PCR analyses showed significant upregulation of the ferritin gene in all tissues. The most pronounced upregulation (5.58- to 9.11-fold) was observed in the digestive gland and mantle of the thick-shell mussel. Furthermore, this study confirmed that ferritin plays a role in protecting against oxidative stress using RNAi gene silencing [54]. Beyond pollutant exposure, natural environmental factors such as temperature and oxidative stress also influence ferritin transcription in the sea snail. In red abalone, ferritin gene expression was upregulated in various tissues under thermal stress conditions (20 °C to 26 °C). This finding suggests that higher temperatures stimulate reactive oxygen species, which modulate ferritin gene regulation in order to suppress Fenton’s reaction. However, elevated temperature affects the whole metabolism of the organism, which might contribute to changes in ferritin gene expression in red abalone [28]. Additionally, the seasonal variations such as temperature, oxidative stress and reproductive cycle influence the natural expression profiles of stress biomarkers in the blue mussel, *M. galloprovincialis* (Lamarck, 1819). The expression of the ferritin gene strongly correlated (*p* < 0.001) with increases in water temperature and decreases in dissolved oxygen. This study further supports the idea that natural environmental stressors influence ferritin expression in bivalve populations [55]. Taken together, these findings align with our observation of upregulated *Mbi-Fer* expression in wild oysters, suggesting that the upregulation of ferritin transcription is a part of the bivalve stress response system against both anthropogenic and natural environmental challenges. Our study emphasizes the idea that *Mbi-Fer* expression might serve as a molecular indicator of environmental and physiological stress in estuarine bivalves.

A highly conserved IRE was identified in the 5′ untranslated region (5′ UTR) of *Mbi-Fer*, located 59 bp upstream of the start codon (spanning from nucleotide positions 15 to 46). The IRE is essential for the translational regulation of ferritin and mediates iron-dependent control of ferritin synthesis [49]. This regulatory mechanism involves an iron-responsive protein that specifically binds to the IRE, thereby modulating ferritin translation based on intracellular iron levels [56]. The IRE identified in the *Mbi-Fer* contains all the structural features required for proper function, including the conserved motif 5′-CAGTGA-3′. Sequence analysis confirmed its identity with IREs from other bivalves, showing 100% similarity to the ferritin GF1 IRE of *M. gigas* (Figure 3), further supporting the conservation of translational regulation mechanisms across oysters.

Mbi-Fer harbors several conserved motifs and amino acid residues that are characteristic of the ferritin protein family and displays significant sequence similarity to ferritin genes from other bivalves, including *M. gigas* and *P. fucata*. Protein structure analysis demonstrated that Mbi-Fer contains conserved residues in the ferroxidase di-iron center (25E, 32Y, 59E, 60E, 63H, 105E, and 139Q) and the iron ion channels (116H, 129D, and 132E), which are characteristic features of ferritin H subunits. In addition, Mbi-Fer also possesses residues of the ferrihydrite nucleation site (58D,59E, and 62E) that were typically found in ferritin L subunits. Therefore, Mbi-Fer could likely be an M-type subunit of ferritin that had structural and functional properties of both H and L subunits. M-type ferritins are commonly found in lower vertebrates such as amphibians and fish. In bivalve molluscs, M-type ferritins have been reported in the Yesso scallop, *Patinopecten yessoensis* (Jay, 1857) [17], and Pacific oyster *M. gigas* [13,21]. In addition to the abovementioned residues, several amino acids conserved in human ferritin were also conserved in Mbi-Fer. These include C128, L112, L136, R74, and D124, which constitute an extended part of the iron ion channel through which Fe^2+^ gains access to the ferritin interior. Furthermore, another group of residues conserved in vertebrate H subunits ferritins (Y27, Y30, Y32, and Y135) was also present in Mbi-Fer, supporting its functional similarity to both vertebrate and invertebrate ferritin types.

Phylogenetic analysis of several vertebrate and invertebrate ferritin sequences revealed that the newly identified *Mbi-Fer* shares significant sequence similarity with ferritin genes from *M. gigas* (CAD92096.1, CAD91440.1, and AAP83793.1), all of which have been classified as M-type ferritin subunits [13]. These findings further support the classification of *Mbi-Fer* identified from *M. bilineata*, as an M-type ferritin subunit. Because the ferritin gene analyzed here is nuclear, it is not affected by doubly uniparental inheritance (DUI), a mitochondrial inheritance system reported in several bivalves. Therefore, whether DUI occurs in *M. bilineata* is unlikely to influence our sequence-based or expression-based results or their interpretation. This agrees with previous studies demonstrating that DUI affects mitochondrial genomes but not nuclear markers [57,58]. An attempt to determine the tertiary structure of Mbi-Fer by homology modeling revealed a typical ferritin structure consisting of five helices (four long and one short). The helices were named A to E from the N (amino) to C (carboxy) terminal end. The short helix (E) present at the C-terminal end is detached from the main tetra-helical structure, which encloses the metal-binding and ferroxidase centers. A long-loop structure connects the two pairs of helices in the tetra-helix bundle [2,11].

We found no signal peptides in the sequence of Mbi-Fer, suggesting that it is likely a cytoplasmic ferritin. This is consistent with most known molluscan ferritins, which are typically localized in the cytoplasm and lack signal peptides. However, there are also a few exceptions, in which signal peptides have been identified in ferritins from *L. stagnalis* [59], *H. discus* [27], and *Biomphalaria glabrata* (Say, 1818) [60]. These exceptions suggest the presence of ferritin isoforms in some mollusks, highlighting the functional diversity of ferritin proteins across species.

## 5. Conclusions

We identified and characterized a ferritin gene from the digestive tissue of *M. bilineata.* Mbi-Fer exhibited all of the structural features that are characteristic of a typical ferritin protein. A phylogenetic study revealed a close evolutionary relationship between *Mbi-Fer* and its invertebrate counterparts, confirming its remarkable identity with other Ostreidae ferritins. The presence of a unique protein band of Mbi-Fer in a recombinant prokaryotic system confirmed the accuracy of its molecular characterization. The upregulation of *Mbi-Fer* in wild oysters indicates that this gene may be useful as a biomarker of oxidative stress in estuarine bivalves. Further study is needed to clarify the expression patterns of *Mbi-Fer* and other stress response genes under varying environmental and pathogenic conditions.

## Figures and Tables

**Table 1:** Environmental parameter variables observed at the time of the Indian backwater oyster sampling. Salinity and temperature were measured in situ, using a salinometer and a precision thermometer, respectively. pH and turbidity were recorded using a pH meter and a digital turbidity meter. Dissolved oxygen concentrations were determined using the modified Winkler titration method. ND: Not detected.

Environmental Parameter	Wild Habitat Site	Laboratory Aquaria
Temperature (°C)	31.0	28.0
pH	7.44	7.42
Salinity (ppt)	15.0	22.0
Turbidity (NTU)	6.60	0.05
Dissolved oxygen (mg/mL)	4.46	5.82
Chlorophyll-a (mg/m^3^)	8.0	ND
NH_4_^+^ (µmoles/L)	2.3	ND
NO_2_−N (µmoles/L)	0.695	ND
NO_3_−N (µmoles/L)	1.2	ND
PO_4_−P (µmoles/L)	0.5	ND

**Table 2:** Sequence comparison of Mbi-Fer with other ferritins (%). This shows homology percentage of Mbi-Fer amino acid sequences with other ferritins.

% Similarity	*M. gigas*	*P. fucata*	*O. edulis*	*H. diversicolor*	*C. concholepas*	*H. sapiens*	*D. rerio*	*M. musculus*
Mbi-Fer	97.66	85.38	84.80	78.95	78.82	66.08	63.53	63.16

## Data Availability

The *Mbi-Fer* sequence has been deposited in GenBank, under the accession number MK820034.

## Supplementary Materials

The following supporting information can be downloaded at: https://www.mdpi.com/article/10.3390/genes17030330/s1. Supplementary Figure S1: Location of the sampling site of the Indian backwater oyster. The red triangle marks the sampling station at the Sathar Island region of the Kodungallur-Azhikode estuary. Supplementary Figure S2: PCR amplification of *Mbi-Fer* and SDS-PAGE analysis of recombinant Mbi-Fer of *M. bilineata*. Lane 1: 100 bp DNA ladder, Lane 2: 516 bp amplified ORF of *Mbi-Fer* gene, Lane 3: uninduced control *E. coli* BL21 cells; Lane 4: induced *E. coli* BL21 cells; Lanes 5 and 6: protein ladder in kDa; and Lane 7: Affinity-purified recombinant Mbi-Fer protein, which appeared as a single band with an approximate molecular weight of ~20 kDa.

## Abbreviations

Mbi-Fer	M-type subunit of ferritin
IRE	iron-responsive element
SDS	sodium dodecyl sulfate polyacrylamide gel electrophoresis
ORF	open reading frame
UTR	untranslated region
