# Supplementary figures and images for: Identification, Cloning and Expression of Ferritin M-like Subunit from the Indian Oyster, Magallana bilineata (Röding, 1798)
Source: Genes (Basel). 2026 Mar 18;17(3):330. doi: 10.3390/genes17030330 (PMC13026281; doi:10.3390/genes17030330)

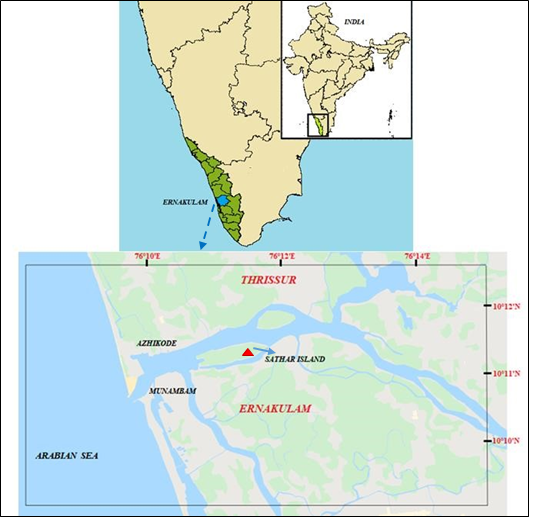

Supplement: Supplementary file 1 [file genes-17-00330-s001.zip › Supplementary Figures/Figure S1.tif]

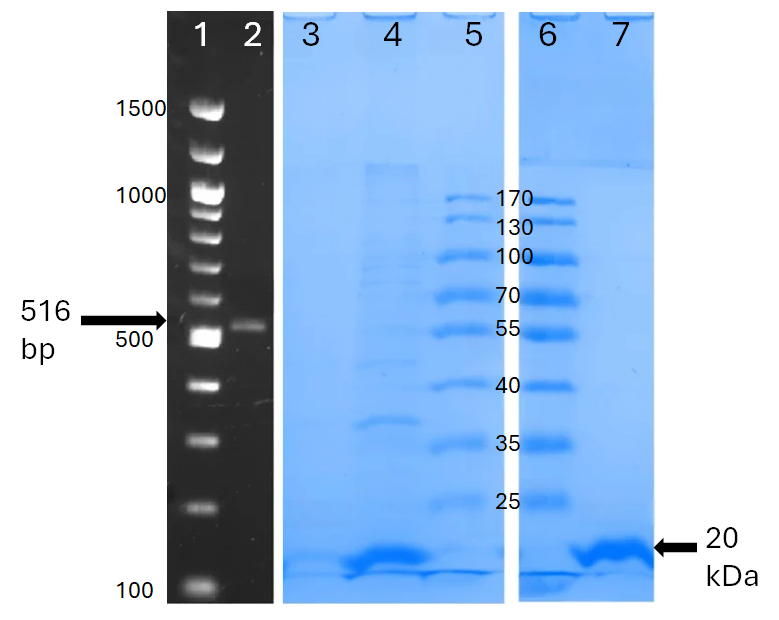

Supplement: Supplementary file 1 [file genes-17-00330-s001.zip › Supplementary Figures/Figure S2.tif]
